# The STING Ligand and Delivery System Synergistically Enhance the Immunogenicity of an Intranasal Spike SARS-CoV-2 Vaccine Candidate

**DOI:** 10.3390/biomedicines10051142

**Published:** 2022-05-16

**Authors:** Tuksin Jearanaiwitayakul, Jitra Limthongkul, Chernkhwan Kaofai, Suttikarn Apichirapokey, Runglawan Chawengkirttikul, Sompong Sapsutthipas, Panya Sunintaboon, Sukathida Ubol

**Affiliations:** 1 Department of Microbiology, Faculty of Science, Mahidol University, Bangkok 10400, Thailand; tuksin.jear@gmail.com (T.J.); jitra.kas@mahidol.ac.th (J.L.); chernkhwan.k@gmail.com (C.K.); suttikarn.api@gmail.com (S.A.); runglawan.cha@mahidol.ac.th (R.C.); 2 Institute of Biological Products, Department of Medical Sciences, Ministry of Public Health, Nonthaburi 11000, Thailand; sompong.s@dmsc.mail.go.th; 3 Department of Chemistry, Faculty of Science, Mahidol University, Salaya 73170, Thailand; panya.sun@mahidol.ac.th

**Keywords:** intranasal COVID-19 vaccine, SARS-CoV-2 spike glycoprotein, adjuvant nanodelivery, STING agonist, immunogenicity

## Abstract

The respiratory organ serves as a primary target site for SARS-CoV-2. Thus, the vaccine-stimulating immune response of the respiratory tract is significant in controlling SARS-CoV-2 transmission and disease development. In this study, mucoadhesive nanoparticles were used to deliver SARS-CoV-2 spike proteins (S-NPs) into the nasal tracts of mice. The responses in the respiratory organ and the systemic responses were monitored. The administration of S-NPs along with cGAMP conferred a robust stimulation of antibody responses in the respiratory tract, as demonstrated by an increase of IgA and IgG antibodies toward the spike proteins in bronchoalveolar lavages (BALs) and the lungs. Interestingly, the elicited antibodies were able to neutralize both the wild-type and Delta variant strains of SARS-CoV-2. Significantly, the intranasal immunization also stimulated systemic responses. This is evidenced by the increased production of circulating IgG and IgA, which were able to neutralize and bind specifically to the SARS-CoV-2 virion and spike protein. Additionally, this intranasal administration potently activated a splenic T cell response and the production of Th-1 cytokines, suggesting that this vaccine may well activate a cellular response in the respiratory tract. The results demonstrate that STING agonist strongly acts as an adjuvant to the immunogenicity of S-NPs. This platform may be an ideal vaccine against SARS-CoV-2.

## 1. Introduction

In December of 2019, a member of the RNA beta coronaviruses emerged. It is known as severe acute respiratory syndrome coronavirus 2 (SARS-CoV-2) [1]. SARS-CoV-2 is the causative agent of the clinical disease called COVID-19. SARS-CoV-2 rapidly spread into 220 countries. This has resulted in at least 507 million confirmed cases and more than 6.2 million deaths, as announced by the World Health Organization (WHO) on 25 April 2022 (https://covid19.who.int/ (accessed on 25 April 2022)). COVID-19 is the third respiratory pandemic caused by infection with a novel coronavirus, with the first and the second ones being SARS (severe acute respiratory syndrome) and MERS (middle east respiratory syndrome), respectively. The severe form of the COVID-19 disease is associated primarily with fever, cough, shortness of breath, and serious lung syndromes, including acute respiratory distress syndrome (ARDS) and cytokine release syndrome (CRS) [2]. The uniqueness of the SARS-CoV-2 infection compared with SARS and MERS is that viral particles are shed during the presymptomatic phase of infection. This has led to the significant spread of the virus worldwide [3]. One approach to stop this pandemic is global immunization with an effective anti-COVID-19 vaccine.

The ideal vaccine against SARS-CoV-2 is a vaccine that acts against infection, disease progression, or transmission [4]. Three platforms of anti-COVID-19 vaccines have recently been launched for human immunization. These are the viral vector vaccines, the nucleic-acid-based vaccines (mRNA vaccine and DNA vaccine), and the non-replicating vaccines (the killed-virus vaccine and the virus-like particle and protein subunit vaccine) [5,6]. The vaccines for emergency use are administered via an intramuscular injection. These vaccines stimulate potent systemic responses against SARS-CoV-2, while specific responses in the primary target organ remain doubtful, as supported by a breakthrough infection by certain genotypes of SARS-CoV-2 in certain vaccinated populations [7]. This may, of course, be due to the continuous evolution of the virus [8] and the sub-neutralizing levels of anti-SARS-CoV-2 responses in the main target organ, the respiratory tract.

The transmission of SARS-CoV-2 mainly occurs via exposure to respiratory secretions and contaminated surfaces, as well as the inhalation of virus particles in the air [9,10]. However, there is also a report of viral shedding through the fecal route [11]. This is evidenced by the presence of the SARS-CoV-2 genome in fecal specimens from COVID-19 patients [12]. Interestingly, spontaneous replication of SARS-CoV-2 was observed in the bacterial cultures of patients’ feces for up to 30 days and beyond [13]. This suggests the association between SARS-CoV-2 and gut microbiota. This may contribute, more or less, to pathogenesis and the mode of transmission.

This virus utilizes angiotensin-converting enzyme 2 (ACE2), which is highly expressed in the nasal epithelium, for its entry [14,15]. The major target organ of SARS-CoV-2 is the respiratory tract; thus, complications of COVID-19 are commonly pulmonary. Therefore, an effective vaccine should stimulate strong protective immunity in the respiratory tract. In other words, a vaccine that is delivered via the intranasal route may be a challenging alternative. Nasal-associated lymphoid tissue (NALT) anatomically located in the nasopharynx and oropharynx area is an immune inductive site and a primary target for intranasal vaccines [16]. An example of an advanced intranasal vaccine for human use is influenza vaccines, including the FluMist^®^ trivalent and quadrivalent seasonal flu vaccines and Nasovac-S [17,18,19]. Both of them are intranasal multivalent vaccines. The FluMist^®^ trivalent vaccine has been approved by US FDA, and is designed to act against two influenza A and one influenza B strains and be able to stimulate strong immunity in individuals aged 2–49 years [17,18]. Nasovac-S is currently licensed in India. This nasal spray is a trivalent vaccine composing of vaccine virus strains of pandemic flu (H1N1) and seasonal flu (H3N2), and influenza B/Victoria lineage. Nasovac-S effectively induces mucosal immune responses and prevents symptomatic illness among children [19,20]. Moreover, its safety and efficacy have been confirmed after administration to a large population, with broad ranges of age [21].

The mucosal epithelial surface is covered with mucus, acting as a physical barrier, and cilia that quickly transport foreign substances out of the body [22]. These innate defense mechanisms can reduce vaccine permeation, resulting in a lower vaccine effectiveness. Thus, solving these problems to increase the efficacy of mucosal vaccines is challenging. To overcome these limitations, nanoparticulate delivery systems have been introduced [23,24,25,26]. This nanocarrier may provide a depot effect, mucoadhesion, and immunostimulatory effects [27,28,29]. Various positively charged mucoadhesive particulate carriers have been investigated, such as those used for hepatitis B or HIV vaccine development [30,31]. They were shown to enhance the immunogenicity of the encapsulated immunogens. To further strengthen the vaccine efficacy, some have combined both immunogens and adjuvants into the nanocarriers [32,33]. This approach can escalate the immunogenicity of the immunogens.

Several studies revealed that the immunogenicity of the inactivated influenza vaccine can be enhanced if an appropriate adjuvant is used [33,34,35]. Our group recently reported that the carbohydrate nanocarrier can escalate the immunogenicity of the spike glycoprotein of SARS-CoV-2 once administered via intranasal immunization as well as intraperitoneal immunization [36,37]. To mimic the responses via the natural route of the SARS-CoV-2 infection, we, here, investigated the immunogenicity of the spike glycoprotein of SARS-CoV-2 administered through an intranasal route. To increase its immunogenicity, the spike glycoprotein was encapsidated in a mucoadhesive nanodelivery platform, and was administered in the presence of a mucosal adjuvant, STING ligand, or 2′,3′-cGAMP. Though cGAMP is a STING ligand, it is hydrolyzed quickly by ecto-nucleotide pyrophosphatase/phosphodiesterase (ENPP1) when located outside the plasma membrane [38]. This ensures the circumvention of unwanted systemic inflammation. This is supported by studies showing that cGAMP does not cause any significant skin or acute local inflammatory responses, and is neither toxic to the liver nor the kidney [39,40]. In our present study, BALB/c mice were intranasally vaccinated with S-NPs + cGAMP. The systemic humoral and cellular responses and the anti-SARS-CoV-2 responses at the respiratory tract were monitored. We confirm here that the delivery system accompanied by cGAMP effectively enhanced the immunogenicity of the spike vaccine in the respiratory tract. Moreover, the immunized mice showed no signs or symptoms of adverse effects.

## 2. Materials and Methods

### 2.1. UV-Inactivated SARS-CoV-2

Two strains of UV-inactivated SARS-CoV-2, wild-type (WT, hCoV-19/Thailand/74/2020) and delta variant lineage B.1.617.2 (CV-2071), were obtained from the Institute of Biological Products, Department of Medical Sciences, Ministry of Public Health, Thailand, and used in capture ELISA.

### 2.2. Animals

Female BALB/c mice (6–8 weeks old) were purchased from Nomura Siam International (Nomura Siam International Co., Ltd., Bangkok, Thailand). The mice were acclimated in an animal facility for a week prior to initial use. All animal protocols in this study were approved and performed in accordance with the Faculty of Science, Mahidol University Animal Care and Use Committee (SCMU-ACUC, protocol number: MUSC63-012-520).

### 2.3. Production of Spike-SARS-CoV-2 Antigen

The full-length spike glycoprotein of SARS-CoV-2 (SARS-CoV-2 GenBank accession number: NC_045512.2) was cloned and expressed by transformed *P. pastoris*, as previously described [37]. The recombinant protein was subsequently purified using affinity chromatography on Ni-NTA agarose (Invitrogen, Carlsbad, CA, USA) [41].

### 2.4. Preparation and Characterization of Spike Loaded in N,N,N-Trimethyl Chitosan Nanoparticles (S-NPs)

The nanoparticle-loaded spike proteins were prepared by ionotropic gelation, as previously described [37]. Briefly, sodium tripolyphosphate (TPP) solution, spike proteins (0.3 mg/mL), and TMC solution in HEPES buffer pH 7.4 were mixed and stirred at room temperature. The spike-encapsidated nanoparticles were separated from the soluble proteins using centrifugation at 10,000× *g* for 10 min. The efficiency of protein loading in TMC NPs was calculated as described previously [42]. The spike-loaded nanoparticles were further characterized using a zetasizer (Malvern Instruments, Malvern, UK).

### 2.5. In Vivo Immunization and Specimen Collection

The mice were intranasally immunized with S-NPs (10 or 20 μg) alone or S-NPs plus 5 μg 2′-3′-cGAMP (Invivogen) or soluble spike glycoprotein (S) at 10 or 20 μg/dose. The mice immunized with 1× PBS, or empty TMC NPs, or cGAMP alone were negative control groups. Vaccination was performed on days 0, 8, 15, and 30 with 20 μL of vaccine per dosage. Blood samples were collected on days 7, 14, and 29. On day 45, blood, BAL, spleens, and lungs were harvested, as previously described [37]. The harvested lung was homogenized in 0.1 mL of PBS containing protease inhibitor cocktail (Roche, Mannheim, Germany), using an electric homogenizer (Argos Technologies^®^ Motorized Pestle Mixer, China), and was then clarified by centrifugation to remove cellular debris. The spleen was harvested, processed to single-cell isolation, and cultured for an assessment of vaccine-induced cell-mediated immune response.

### 2.6. Quantitation of Antibody Titers

The levels of SARS-CoV-2 spike-specific IgG, IgG1, IgG2a, and IgA isotype antibodies present in the serum, BAL, and lung homogenate were measured using indirect ELISA, as previously described [36]. Briefly, 96-well microplates were pre-treated with purified spike protein antigens (1 μg/mL) at 4 °C for 16 h, washed, and blocked with blocking buffer. The antigen-coated plates were incubated with 100 μL of two-fold serial dilutions of the samples (sera, BALs, and lung homogenates). Antigen–antibody interactions were detected using goat anti-mouse IgG (1:3000, Invitrogen), IgG1, IgG2a, or IgA antibody-labeled horseradish peroxidase (HRP) (1:4000, Southern Biotech, Birmingham, AL, USA) and the TMB substrate. The absorbance was then read at 450 nm using an ELISA reader. The cut-off of endpoint titers (EPT) of the spike-specific antibodies were determined by the reciprocal of the highest dilution of samples given an absorbance above the three-fold OD value of blank control [36].

### 2.7. Whole-Virion Capture IgG and IgA ELISAs

The level of antibody specific to SARS-CoV-2 was quantitated using virion capture ELISA, as previously described [36]. The plates were pre-coated with rabbit SARS-CoV-2 polyclonal antibody (1:4000, Sino Biological, Beijing, China) overnight at 4 °C, washed, and blocked with 1% BSA in PBST. UV-inactivated SARS-CoV-2 (10⁴ PFU/well) of WT or Delta strain diluted in MEM medium was added into each well and incubated for 2 h. After incubation, diluted samples of immunized mice were applied and incubated. The levels of anti-SARS-CoV-2 antibody were detected using HRP-labeled goat anti-mouse IgG (1:3000, Invitrogen) or IgA antibody (1:4000, Southern Biotech, Birmingham, AL, USA), respectively. The plates were washed and TMB substrate was added for signal development. The reactions were terminated using 1 N HCl, and the absorbances were recorded at 450 nm. 

### 2.8. In Vitro Virus Neutralization Assay

The level of neutralizing antibody (NAb) was determined using plaque reduction neutralization assay (PRNT). Briefly, mouse sera were 4-fold diluted and mixed with an equal volume of 10^2^ PFU of SARS-CoV-2 at 37 °C for 1 h before being transferred into a monolayer of Vero cells. The viruses were allowed to adsorb at 37 °C for 1 h with gentle rocking. Viruses incubated with medium were used as a control. The plaque assay was performed as recently described [36]. The number of plaques was counted and the percentage of plaque reduction at 50% was then calculated compared with the virus control (virus alone).

### 2.9. Surrogate Viral Neutralization Test (sVNT)

The surrogate virus neutralization present in the BALs and lung homogenates was analyzed using sVNT, according to the manufacturer’s instructions (cPass™, GenScript, Piscataway, NJ, USA). Briefly, HRP-labeled RBD was pre-incubated with diluted samples at 37 °C for 0.5 h. After incubation, the mixtures were then transferred into hACE2 pre-coated plates, and further incubated at 37 °C for 15 min. After washing, bound RBD-ACE2 was detected by adding 100 μL of TMB solution. The reaction was stopped by adding 50 μL of stop solution, and signals were read at 450 nm. Percent inhibition was calculated by comparing the OD values of the sample and the negative control.

### 2.10. Detection of Spike-Specific IgA-Secreting Cells

The number of spike-specific IgA-secreting cells was enumerated by ELISPOT assay, as described [34]. Briefly, MultiScreen IP filter plates (96-well) (Millipore, Billerica, MA, USA) were coated with spike protein (2 μg/well) in PBS at 4 °C overnight, before being blocked with 10% FBS containing RPMI 1640 medium (Gibco) for 2 h at room temperature. Afterward, three-fold dilutions of splenocytes were started from 4 × 10^6^ cells/well, loaded into each well and cultivated at 37 °C with 5% CO_2_ for 16 h. After incubation, the cells were removed and the plates were washed with PBST three times. To detect the IgA-secreting cells, HRP-labeled goat anti-mouse IgA (Southern Biotech, Birmingham, AL, USA) was added and incubated for 1 h at room temperature. The signals were developed by staining with DAB (SigmaFast DAB tablet, Sigma-Aldrich, St. Louis, MO, USA). The spots were scanned and counted on an ImmunoSpot^®^ S6 Ultimate Reader.

### 2.11. Ex Vivo Stimulation of Splenic Lymphocytes

Briefly, splenocytes (10^7^ cells/well) were cultivated in the presence of spike (10 μg/mL) as a specific antigen. An unstimulated treatment was used as a negative baseline of stimulation. The stimulated cells were grown at 37 °C for 72 h of the stimulation period. After incubation, the cells were harvested, incubated with TruStrain FcX (anti-mouse CD16/32 antibody, BioLegend, San Diego, CA, USA), and subjected to staining with antibodies specific to CD3, CD4, and CD8 (BD Biosciences, San Diego, CA, USA). The stained cells were subsequently analyzed using flow cytometry.

Simultaneously, the culture supernatant was harvested on days 1, 2, and 3. The secreted cytokines, including IFN-γ, IL-2, and IL-4, were monitored using an ELISA assay (BioLegend).

### 2.12. Statistical Analysis

The results were presented as mean ± standard deviation (SD). Statistical analysis was conducted using a *Student’s t*-test for comparison between the two groups of study. A *p* value < 0.05 was considered as a statistical difference.

## 3. Results

### 3.1. Construction and Characteristics of Spike Nanoparticles (S-NPs)

S-NPs were prepared using the ionic gelation method. After NP fabrication, as high as 95% of S-proteins were successfully encapsulated in trimethyl chitosan nanoparticles (TMC NPs). Dynamic light scattering analysis revealed that the S-NPs were 343 ± 3.4 nm in diameter with a positive net charge (ζ = +14.9 ± 0.541).

### 3.2. S-NPs Adjuvanted with STING Agonist Effectively Induce Antibody Responses at Respiratory Airways

**Figure 1:**
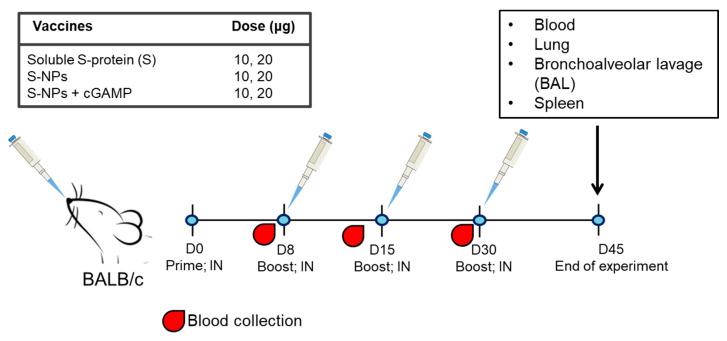
Schematic diagram of immunization and sample collection. Mice were administered intranasally with soluble S-protein, S-NPs, or S-NPs + cGAMP at 10 or 20 μg/dose. Prime immunization was performed on day 0, followed by three booster shots on days 8, 15, and 30. Sera were collected from immunized mice on days 7, 14, and 29. On day 45 post-immunization, mice were euthanized and samples including blood, lung, BAL, and spleen were harvested for assessment of humoral and cell-mediated immune responses.

To evaluate the safety and immunogenicity of our vaccine formulations, BALB/c mice were nasally inoculated with 10 or 20 μg/dose of the following tested immunogens: a soluble S-protein (S), S-NPs alone, or S-NPs plus cGAMP (S-NPs + cGAMP) (Figure 1). Vaccinations were performed on days 0, 8, 15, and 30. The mice were routinely assessed for weight loss and health status. There was no alteration in their body weight or any adverse effects seen between the groups of mice vaccinated with the soluble S-protein, S-NPs, or S-NPs + cGAMP, or the negative controls across the time course of the observation (data not shown).

**Figure 2:**
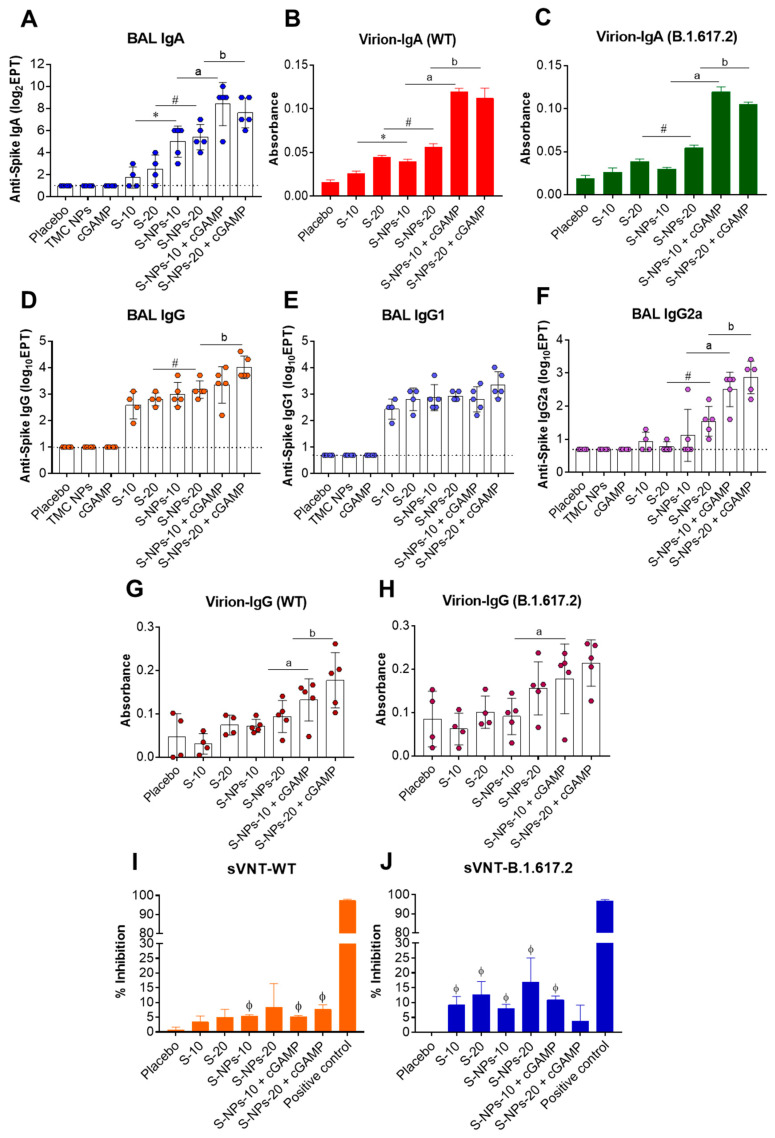
Mucosal immune responses in BALs of immunized mice. BALs were collected from immunized mice on day 45. S-IgA titers were determined by indirect ELISA (**A**). The levels of WT virion-IgA (**B**) and Delta variant (B.1.617.2) virion-IgA (**C**) in pooled BALs at 1:2 dilution were measured using virion-IgA capture ELISA with three independent experiments. Levels of spike-specific IgG (**D**), IgG1 (**E**), and IgG2a (**F**) antibodies were quantitated by ELISA. BALs at a dilution of 1:10 were analyzed for WT virion-IgG (**G**) and Delta variant virion-IgG (**H**) capture ELISA assays. The potential neutralizing activity against WT (**I**) and Delta variant (**J**) in BALs at a final dilution of 1:2 was monitored by surrogate virus neutralization assay (sVNT). Results are shown as mean ± SD. ᶲ indicates significant differences between S-containing vaccines and placebo. * and # indicate significant differences between soluble S-protein and S-NPs at 10 and 20 μg/dose, respectively. “a” and “b” indicate significant differences between S-NPs and S-NPs + cGAMP at 10 and 20 μg/dose, respectively (*p* < 0.05). Dotted line indicates LoD of the assay.

cGAMP has previously demonstrated its potency as a mucosal adjuvant through intranasal immunization, resulting in an exacerbation of mucosal IgA responses [35,43]. As the respiratory tract is the entry port of the SARS-CoV-2 infection, we therefore determined how well our vaccine platform activates mucosal immunity in the respiratory tract. On day 45, the lungs were harvested and the airway spaces were flushed with 1 mL of 1× PBS to obtain bronchoalveolar lavages (BALs). The harvested BALs were subjected to IgG/IgA quantification. As shown in Figure 2A, S-specific IgA responses were stimulated in the BAL of all mice treated with S-NPs or S-NPs + cGAMP. Meanwhile, 2/4 and 3/4 of the mice immunized with the soluble S-proteins at 10 or 20 μg/dose, respectively, elicited detectable levels of secretory IgA (sIgA). As expected, S-NPs activated a stronger IgA response in the BALs than the soluble S-protein did. Furthermore, we observed a remarkably high concentration of IgA in the BALs of mice immunized with S-NPs adjuvanted with a STING agonist (Figure 2A). Consistent with the results of S-binding IgA, IgA antibodies present in the BALs of S-NPs + cGAMP-immunized mice efficiently bound to SARS-CoV-2 virus particles from both the WT and Delta isolates (Figure 2B,C).

The productions of anti-S IgG in BALs were significantly increased upon intranasal immunization with either the soluble S-protein, S-NPs alone, or S-NPs with cGAMP, compared with the mice immunized with control compounds (Figure 2D). As expected, the mice that received S-NPs + cGAMP elicited stronger BAL IgG responses than the mice immunized with the soluble S-protein or S-NPs at the same concentration. To profile the IgG subtypes of the antibodies presented in the BALs, IgG1 and IgG2a antibodies specific to the S-protein were determined by indirect ELISA. Figure 2E shows that intranasal administration with the soluble S-protein, S-NPs, or S-NPs + cGAMP resulted in elevated BAL IgG1 levels. However, S-NPs + cGAMP stimulated BAL IgG1 production at similar levels to the soluble S-protein and S-NPs (Figure 2E). In contrast, the mice administered with S-NPs + cGAMP elicited a robust BAL IgG2a response (Figure 2F). The results suggest that cGAMP synergized with S-NPs to promote IgG2a responses toward the S-protein at the mucosal sites. Moreover, we found that the BAL IgG antibodies induced by the S-NPs + cGAMP vaccination strongly interacted with native epitopes on the SARS-CoV-2 virion derived from both the WT and the Delta variant (Figure 2 G,H). 

The efficiency of the antibody responses in the BALs to inhibit the engagement of the receptor-binding domain (RBD) of SARS-CoV-2 and ACE2 was further investigated by a surrogate neutralization assay. We found that BALs of the mice that received either soluble-S protein, S-NPs, or S-NPs + cGAMP had a higher ability to inhibit the engagement between RBD and ACE2 than the BALs of the placebo mice did (Figure 2I,J). However, there were no significant differences in the inhibitory activity observed among the groups of mice receiving immunogens. This may be due to our finding that all groups of mice that received immunogens developed anti-RBD IgA and IgG in their BALs at a comparable level (data not shown).

### 3.3. Antibody Responses Induced by Immunization with S-NPs + cGAMP in Lung Tissues

**Figure 3:**
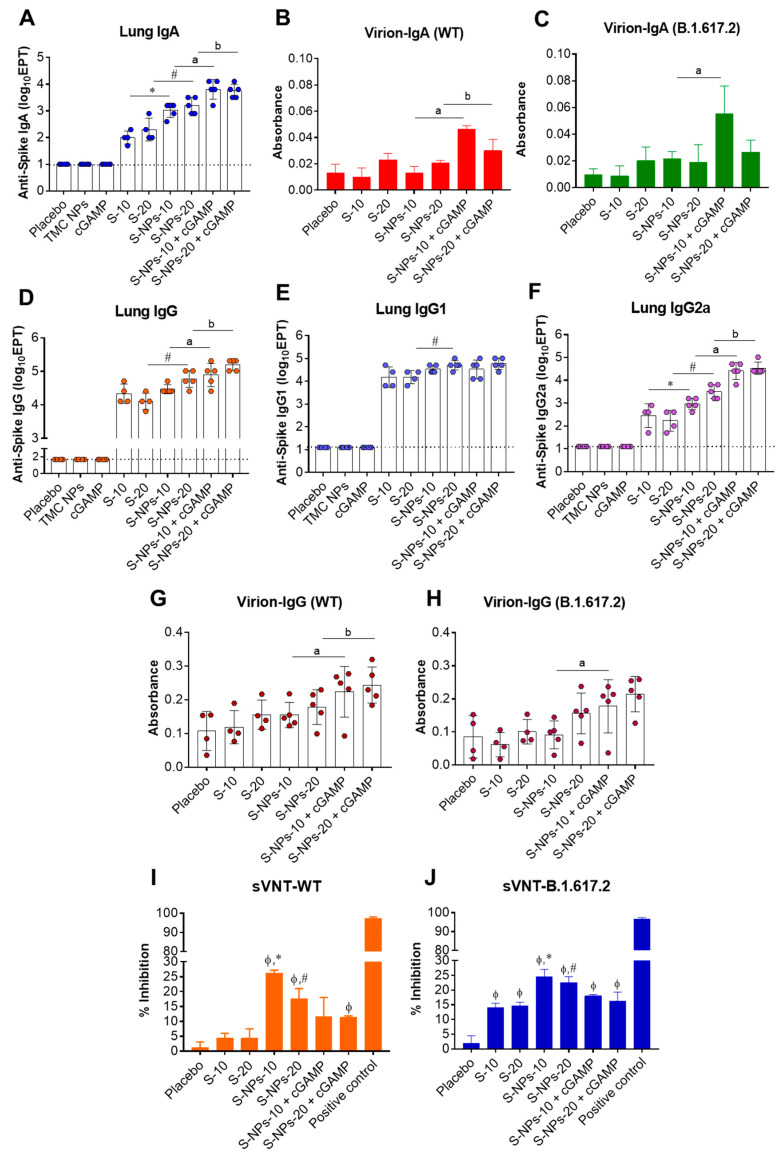
Mucosal immune responses in lung tissues of immunized mice. Mice were intranasally administered with four doses of soluble S-protein (S), S-NPs, or S-NPs + cGAMP. On day 45 post-immunization, lungs were harvested and processed by homogenization. Lung homogenates were subjected for quantitation of S-IgA titers by indirect ELISA (**A**). Levels of WT virion-IgA (**B**) and Delta variant virion-IgA (**C**) in pooled lung homogenates at 1:10 dilution were determined by virion-IgA capture ELISA. Lung homogenates were analyzed for S-IgG (**D**), S-IgG1 (**E**), and S-IgG2a (**F**) titers by ELISA. Binding of antibodies present in lung homogenates at a dilution of 1:50 to SARS-CoV-2 WT (**G**) or Delta variant virion (**H**) was detected by virion-IgG capture ELISA. Potential neutralizing activity against SARS-CoV-2 WT (**I**) and Delta variant (**J**) of antibodies present in pooled lung homogenates at a final dilution of 1:2 was determined by surrogate virus neutralization assays (sVNT). Results are presented as mean ± SD. ᶲ indicates significant differences between S-containing vaccines and placebo. * and # indicate significant differences between soluble S-protein and S-NPs at 10 and 20 μg/dose, respectively. “a” and “b” indicate significant differences between S-NPs and S-NPs + cGAMP at 10 and 20 μg/dose, respectively (*p* < 0.05). Dotted line indicates LoD of the assay.

Beyond the antibodies located in the airway spaces of the bronchioalveolar cavity, a certain proportion of antibodies and antibody-secreting cells, particularly memory-resident B cells, localizes in lung tissues (interstitial tissue) in response to respiratory infections [44,45]. This information motivated us to study antibody production in the lung tissue compartment. The lungs of immunized mice were harvested and subjected to homogenization on day 45. The levels of anti-S IgA and IgG antibodies present in lung homogenates were quantitated using indirect ELISA. Figure 3A shows that S-IgA responses could be efficiently induced in the lungs of all mice treated with either the soluble S-protein, S-NPs, or S-NPs + cGAMP. At the same dose of the S-protein, S-NPs significantly mounted a higher lung IgA response than the soluble proteins did. The lung IgA production was remarkably increased by the S-NPs + cGAMP administration compared with stimulation with S-NPs alone (1200 ± 566 vs. 8000 ± 4800, and 1920 ± 1213 vs. 6400 ± 3919 at 10 and 20 μg of S-NPs and S-NPs + cGAMP, respectively) (Figure 3A). Similarly, S-NPs + cGAMP-immunized mice produced lung S-IgA with a strong binding activity against the virion surface of both the WT and the Delta variant (Figure 3B,C).

A similar trend of data was illustrated for S-specific IgG production, in which S-NPs and S-NPs + cGAMP robustly stimulated S-IgG in lung tissues (Figure 3D). In particular, cGAMP-adjuvanted S-NPs activated S-IgG responses more efficiently than S-NPs did (30,720 ± 11,449 vs. 97,280 ± 68,692, and 66,560 ± 34,346 vs. 163,840 ± 56,087 at 10 and 20 μg of S-NPs and S-NPs + cGAMP, respectively). IgG subclasses in lung tissues were also studied using indirect ELISA. The results showed that an increase in S-IgG1 was demonstrated in the lung tissues of all mice treated with the soluble S-protein, S-NPs, or S-NPs + cGAMP (Figure 3E). Only S-NPs at 20 μg significantly induced a lung S-IgG1 response greater than that of the soluble S-protein at the same concentration. Similar to S-IgG1 in the BALs shown above, lung IgG1 titers of S-NPs + cGAMP-immunized mice were found in a similar magnitude to those shown in mice treated with S-NPs. Contrarily, IgG2a titers were significantly higher in the lungs of the mice vaccinated with S-NPs + cGAMP compared with the mice treated with S-NPs (Figure 3F). We next investigated the potency of induced S-IgG antibodies to interact with epitopes on the SARS-CoV-2 virion using capture ELISA. As expected, the antibodies present in the lung homogenate of S-NPs + cGAMP-treated mice were able to react with SARS-CoV-2 particles from the WT, as well as the Delta isolates, stronger than the antibodies from the soluble S-protein or S-NP-immunized mice reacted (Figure 3G,H).

The potential neutralizing activity present in the antibodies produced in lung tissues was determined by a surrogate neutralization assay. As depicted in Figure 3I,J, immunization with any of the tested forms of the immunogen stimulated the production of the antibody that inhibits the interaction between RBD and ACE2 more than the placebo immunization did. Among the tested formulations, the antibodies harvested from the mice receiving S-NPs at 10 μg exhibited the highest surrogate neutralizing activity, with an average of 26.14% and 24.58% neutralization against the WT strain and the Delta variant, respectively (Figure 3I,J).

Taken together, the antibody responses found in the airway cavities and lung tissues of S-NPs + cGAMP-immunized mice indicate that the delivery system synergized with the STING agonist in order to enhance the responses toward an IgG2a profiling.

### 3.4. Intranasal Immunization with S-NPs + cGAMP Strongly Activated Systemic Humoral Immunity

**Figure 4:**
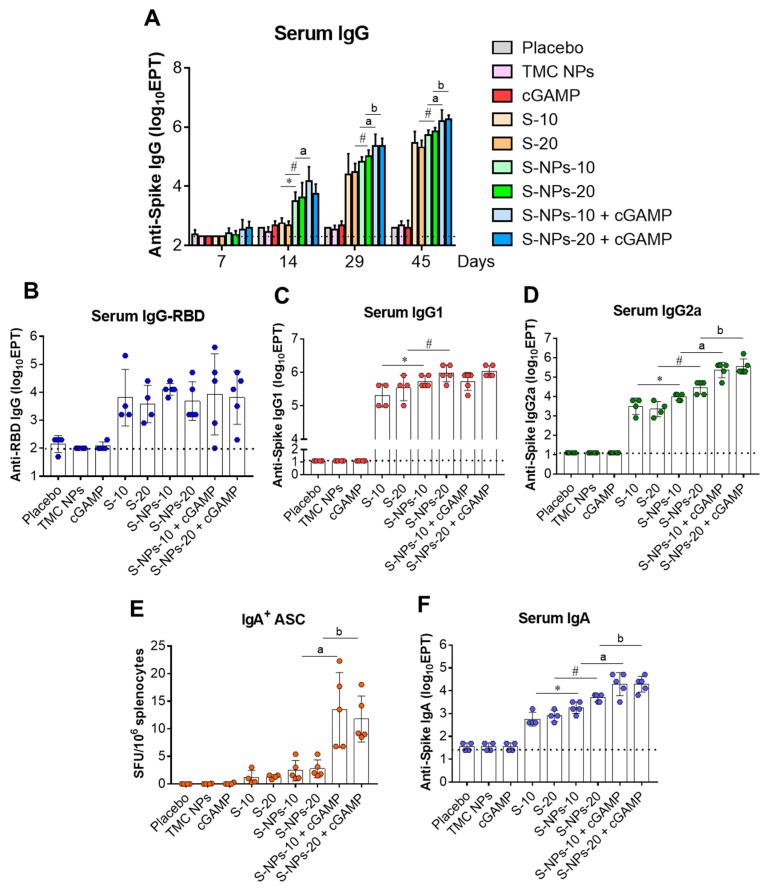
Systemic antibody responses induced by soluble S-protein, S-NPs, or S-NPs + cGAMP. Mice were administered intranasally with a four-dose regimen of soluble S-protein (S), S-NPs, or S-NPs + cGAMP at 10 or 20 μg/dose. Sera collected from immunized mice on days 7, 14, 29, and 45 were analyzed for S-IgG ELISA assay (**A**). IgG titers against RBD protein (**B**), S-IgG1 (**C**), and S-IgG2a (**D**) in sera on day 45 were determined by indirect ELISA. Frequencies of S-specific IgA^+^ B cells in spleen were enumerated by ELISPOT (**E**). Levels of IgA in immunized sera on day 45 were quantitated by indirect ELISA (**F**). Data are presented as mean ± SD. * and # indicate significant differences between soluble S-protein and S-NPs at 10 and 20 μg/dose, respectively. “a” and “b” indicate significant differences between S-NPs and S-NPs + cGAMP at 10 and 20 μg/dose, respectively (*p* < 0.05). Dotted line indicates LoD of the assay.

We investigated whether immunization via the intranasal route can stimulate systemic anti-SARS-CoV-2 responses. The kinetics of anti-S IgG responses in the sera of immunized mice on days 7, 14, 29, and 45 were studied by indirect ELISA. As shown in Figure 4A, on day 7 or after receiving one dose of immunogen, a low level of anti-S IgG was detected in the mice immunized with S-NPs + cGAMP, but not in the other groups of mice. After two doses of vaccination, the S-NPs + cGAMP-immunized mice produced significantly higher anti-S IgG titers than the mice in other groups. The production of anti-S IgG in all the groups of mice that received spike immunogen continuously increased up to the end of our study (day 45). Among the groups that received immunogens, S-NPs + STING agonist stimulated the highest levels of anti-S IgG (2,048,000 ± 1,228,800 and 1,966,080 ± 732,715 for 10 and 20 μg/dose of the encapsidated S-protein, respectively) (Figure 4A). In contrast, mice receiving a soluble form of the immunogens generated significant levels of anti-S IgG on day 29 or after receiving three doses of the vaccination. This indicated a significant delay in antibody stimulation by the soluble S-proteins compared with the encapsidated S-protein at the same concentration.

The amount of anti-RBD antibody in the serum of COVID-19 patients is reported to positively correlate with the anti-SARS-CoV-2 neutralizing activity [46]. We therefore monitored the levels of anti-RBD antibodies in the mice that received different forms of immunogen compared with the control groups using an RBD-IgG ELISA assay. The results showed that a significant increasing in anti-RBD antibodies was found in the sera of mice that received the soluble S-protein, S-NPs, or S-NPs + cGAMP compared with mice in the control groups (Figure 4B). Surprisingly, there was no significant difference in the anti-RBD IgG levels observed between the soluble S-protein and encapsulated immunogens (Figure 4B).

On day 45, sera from the vaccinated mice were determined for S-specific IgG1 and IgG2a profiling using indirect ELISA. As illustrated in Figure 4C,D, intranasal immunization with either soluble-S protein, S-NPs, or cGAMP-adjuvanted S-NPs could lead to an activation of IgG1 and IgG2a production. As expected, the titers of both IgG isotypes stimulated by the S-NPs were significantly higher than those activated by a soluble form of antigen. While S-NPs alone or S-NPs + cGAMP immunization yielded a similar level of serum IgG1 titers, S-NPs + cGAMP vaccination significantly augmented serum IgG2a titers compared with S-NP immunization (Figure 4C,D). The results suggested that the S-protein, by itself, stimulates both IgG1 and IgG2a responses, while the adjuvant activity of cGAMP promotes the IgG2a response.

IgA is the first immunoglobulin detected in blood circulation during the acute phase response against the SARS-CoV-2 infection, presumably a frontline systemic defense to this novel virus [46,47]. Thus, the ability of the vaccines to stimulate a systemic IgA response was investigated. By day 45, the frequencies of the S-specific IgA^+^-producing cells in the spleens of the immunized mice were quantitated using ELISPOT. The results showed that the activation of the IgA⁺ cells could be observed in the mice immunized with either the soluble S-protein, S-NPs, or S-NPs + cGAMP. Interestingly, S-NPs + cGAMP receiving mice elicited the greatest number of IgA⁺ splenic lymphocytes, whereas the mice treated with the soluble S-protein or S-NPs generated a significantly lower level of IgA⁺-producing splenic cells (Figure 4E). To validate the vaccine-induced systemic IgA response, the anti-S IgA in the sera obtained on day 45 were measured using indirect ELISA. As expected, S-NPs + cGAMP significantly increased the serum IgA response compared with S-NPs (Figure 4F). This positively correlated with an increasing frequency of S-specific IgA^+^-producing cells. These observations indicated that intranasal vaccination with S-NPs combined with cGAMP adjuvant potently activates systemic IgA responses.

### 3.5. Functional Capacity of Spike-Specific Antibodies against SARS-CoV-2

**Figure 5:**
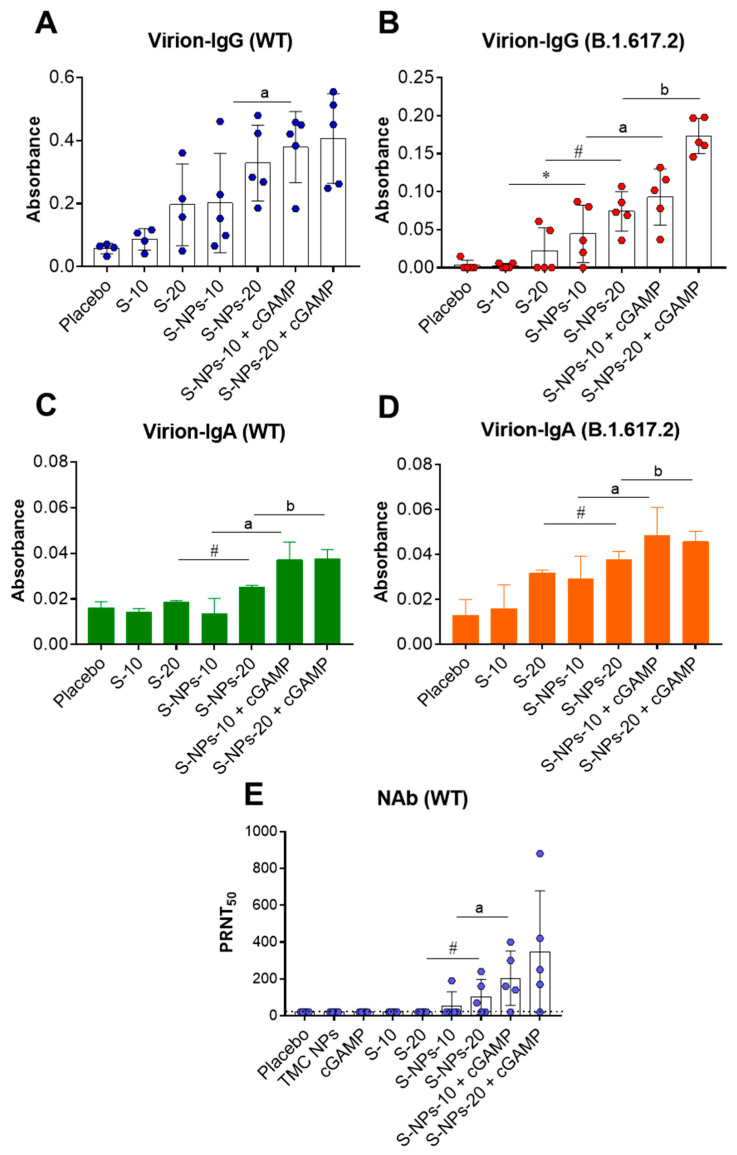
Anti-viral activity of anti-SARS-CoV2 spike antibodies. Sera of immunized mice 2 weeks after last booster at a final dilution of 1:50 were tested for virion-IgG ELISA assay against WT (**A**) and Delta virus variant (**B**). Pooled sera at 1:10 dilution were performed by virion-IgA ELISA assay against WT (**C**) and Delta variant (**D**) with three independent experiments. Neutralizing antibody titers were determined by PRNT_50_ with live WT virus (**E**). Data are presented as mean ± SD. * and # indicate significant differences between soluble S-protein and S-NPs at 10 and 20 μg/dose, respectively. “a” and “b” indicate significant differences of S-NPs compared with S-NPs + cGAMP at 10 and 20 μg/dose, respectively (*p* < 0.05). Dotted line indicates LoD of the assay.

To investigate the efficiency of the antibodies from the immunized mice on interactions with native epitopes on the viral particles, sera obtained on day 45 were subjected to IgG and IgA capture ELISAs. As depicted in Figure 5A,B, antibodies from the mice immunized with either S-NPs or S-NPs + cGMPs strongly interacted with epitopes on the UV-inactivated virions of WT and Delta SARS-CoV-2. Notably, the S-NPs + cGAMP-treated mice had serum IgG antibodies that reacted with both strains of SARS-CoV-2 stronger than sera of the mice administered with S-NPs alone (Figure 5A,B). A similar trend of results was demonstrated for virion-binding IgA, in which the mice immunized with S-NPs + cGAMP exerted the strongest binding capacity against SARS-CoV-2 from both virus strains (Figure 5C,D).

We next tested the potential of SARS-CoV-2-binding antibodies in neutralizing the virus infection. An in vitro neutralization assay (PRNT) was performed using live WT SARS-CoV-2. Figure 5E showed that 1/5 and 3/5 of the mice that were given S-NPs at 10 or 20 μg/dose, respectively, elicited a detectable level of virus-neutralizing antibodies (NAb) in their sera, while 4/5 of the mice immunized with S-NPs + cGAMP at 10 or 20 μg/dose developed a NAb response. As expected, none of the mice that received a soluble form of the S-protein contained detectable levels of NAb (Figure 5E). Taken together, our findings demonstrate that intranasal delivery of S-NP in the presence of the cGAMP adjuvant stimulates a robust systemic antibody response with neutralizing activity against SARS-CoV-2.

### 3.6. Intranasal Administration of S-NPs + cGAMP Induces a Splenic Cell-Mediated Immune Response

**Figure 6:**
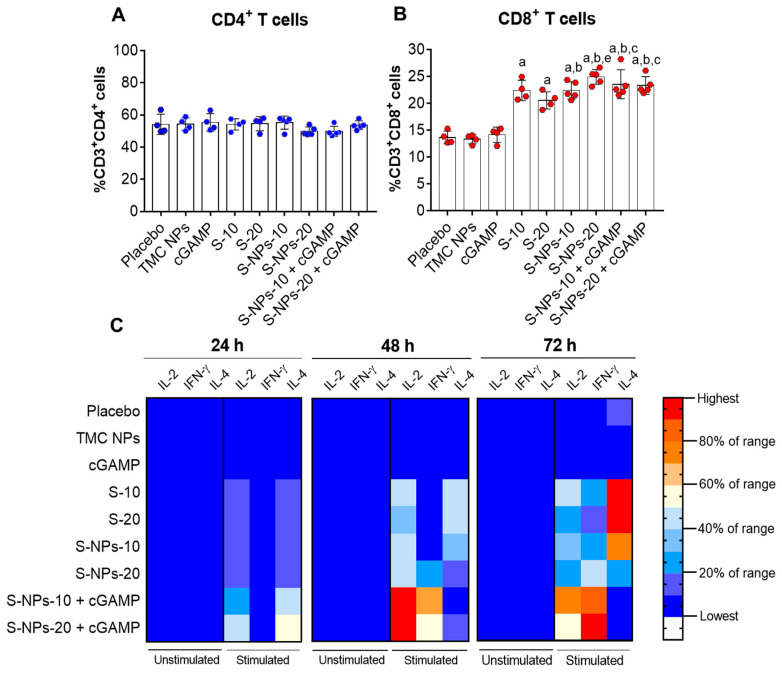
Systemic T cell responses. Splenocytes from immunized mice on day 45 were isolated and restimulated with S-protein (10 μg/mL). After 72 h post-treatment, splenocytes were harvested and subjected to measuring the percentages of CD4^+^ (**A**) and CD8^+^ (**B**) T cells by flow cytometry. Results are mean ± SD. (**C**) Heat map representing splenic cytokine profiles (IL-2, IFN-γ, and IL-4) from culture supernatant at three consecutive days (24, 48, and 72 h of treatment). “a” indicates significant differences between S-containing vaccines and placebo group. “b” indicates significant differences of S-NPs or S-NPs + cGAMP compared with TMC NPs. “c” indicates significant differences between S-NPs + cGAMP and cGAMP adjuvant. “e” indicates significant differences between soluble S-protein and S-NPs at 20 μg/dose (*p* < 0.05).

Aside from antibody-mediated protection, T cells are a part of the host defense in response to viral pathogens. T cells are typically involved in viral clearance via the direct killing of the infected cells, potentiating antibody production and promoting the effector function of killer T cells [48]. Recent studies have shown that SARS-CoV-2-specific CD8⁺ T cells are found to associate with the mitigation of disease burden [49,50], supporting the importance of cellular immunity in controlling the SARS-CoV-2 infection. These observations motivate us to explore the potential effect of S-NPs + cGAMP on the elicitation of cellular immunity. Mice were vaccinated intranasally with a four-dose regimen of the soluble S-protein, S-NPs, or S-NPs + cGAMP. Two weeks after the last booster shots, the spleens were harvested, and the splenocytes were isolated and stimulated with an S-antigen. The frequencies of the CD4⁺ and CD8^+^ cells were then enumerated using flow cytometry. As shown in Figure 6A, intranasal immunization with a soluble or particulate form of the S-protein (with or without cGAMP) yielded a comparable percentage of CD4⁺ T cells compared with negative control groups (placebo, TMC NPs, and cGAMP). The differences were found in the percentage of CD8^+^ T cells. As demonstrated in Figure 6B, all the mice that received the soluble S-protein, S-NPs, or S-NPs + cGAMP developed greater CD8⁺ T cell responses than that of the negative control groups. Interestingly, S-NPs at the 20 μg/dose significantly augmented CD8⁺ T cell expansion compared with the soluble S-protein at the same concentration, while S-NPs + cGAMP stimulated the CD8⁺ T cell population at a similar level to S-NPs. Our findings indicated that the SARS-CoV-2 spike antigen is highly immunogenic to CD8^+^ T cells.

To further support the induction of systemic cellular immunity, the splenocytes from the immunized mice were cultured and stimulated with the S-protein. The levels of IL-2, IFN-γ (Th-1 cytokine), and IL-4 (Th-2 cytokine) in the supernatant were quantitated. Figure 6C and Figure S1A revealed that increasing levels of IL-2 were detected at 24 h of treatment in the mice that received either the soluble S-protein, S-NPs, or S-NPs + cGAMP. Among these forms of immunogens, S-NPs + cGAMP activated the highest IL-2 production. The IL-2 level peaked at 48 h and was maintained at a high level to the end of experimentation (Figure 6C and Figure S1A). The stimulation of IFN-γ followed a similar profile to the IL-2 production, in which splenocyte cultures of S-NPs + cGAMP-treated mice significantly upregulated IFN-γ production within 24 h of cultivation. IFN-γ production was gradually increased and peaked at 72 h post-treatment (Figure 6C and Figure S1B). In contrast, IL-4 production was transient and at a much lower level than IL-2 and IFN-γ production. Accordingly, these results suggested an immune enhancement toward a Th-1 cytokine profile. Overall, the results from the percentage of CD4⁺/CD8⁺ T cells and splenic cytokine profiles indicated that S-NPs adjuvanted with the STING agonist potently activate the cell-mediated immune response.

## 4. Discussion

To date, all authorized SARS-CoV-2 vaccines for human emergency use are based on the spike protein, a major component of the SARS-CoV-2 envelope. These vaccines are given via intramuscular injection [51]. Unfortunately, vaccines administered via this route provide marginal protection against nasal virus replication due to the lack of strong local responses in the nasal cavity [52,53]. As a consequence, vaccine-induced sub-optimal immunity at virus target organs may create a selective pressure for generating newly emerging variants that possess strengthened viral transmissibility and immune escape [54]. To this end, intranasal vaccination that mimics the natural SARS-CoV-2 infection may have more advantages than intramuscular vaccination [55]. Nasal vaccination is believed to stimulate profound mucosal immunities consisting of antibodies and local T cells in the respiratory tract that lead to reduced nasal viral shedding, a blocking of disease development, and, finally, a stimulation of herd immunity [52,56].

In the current study, we found that mice that were nasally administered with S-NPs developed robust mucosal and systemic anti-SARS-CoV-2 responses. For mucosal responses, sIgA is one of the key host immune components, endowing an early defense against pathogens and maintaining homeostasis at the mucosal surface [57,58]. Hassan et al. reported that mice that were intranasally immunized with ChAd-SARS-CoV-2-S not only strongly produced IgA in BALs, but were also completely protected from a nasal SARS-CoV-2 infection [52]. This suggests the pivotal function of at least sIgA in preventing the SARS-CoV-2 infection in the upper respiratory tract. In COVID-19 patients, IgA responses with a high neutralizing potency appeared earlier than that of IgG and IgM responses [46,47]. This suggests that IgA may have a significant role in protecting against SARS-CoV-2 in the acute stage of infection. We demonstrated, here, that S-NPs drastically activated IgA and IgG production in BALs and lung tissues. These antibodies strongly bound to native antigens on the virions, and also exerted neutralizing activity, which was detected using a surrogate neutralizing assay. Whether this sIgA can prevent nasal invasion by SARS-CoV-2 is deserving of further investigation in an in vivo challenge model. Moreover, S-NPs were capable of inducing homo- and heterotypic virion-binding antibodies to a greater extent than the soluble form of the spike proteins did. These results imply that, beyond enhancing the immunogenicity of immunogens, TMC NPs may enable the folding of the spike protein into a conformation that mimics those native structures present on the virion surface.

To further enhance the immunogenicity of our nasal vaccine candidate, cGAMP was used as an adjuvant. As the cGAMP is a STING ligand, it thus orchestrates the cascades of the interferon regulatory factor (IRF-3)- and NF-kβ-dependent pathways, resulting in the release of cytokines, such as Type I interferons and Th-1 cell-recruiting chemokines [39,59]. In addition, through intranasal administration, cGAMP promotes IgA^+^ B cell proliferation, T cell responses, and germinal center (GC) formation at NALTs [60]. Therefore, the interaction of cGAMP with those principal immune cells at NALTs is likely to be critical for the development of potent innate and long-lasting antibody and T cell responses [61]. Based on these immune-adjuvant activities, cGAMP has been applied in different vaccine formulations against respiratory infectious diseases. For instance, cGAMP nasally co-administered with an inactivated influenza vaccine potently evokes humoral, cellular, and mucosal immune responses. This also provides broad-spectrum protection against influenza viruses challenging with corresponding subtype and heterosubtypic strains [33,35]. More recently, using cGAMP and flagellin as a nasal adjuvant alongside combined antigen SARS-CoV-2 proteins (nucleocapsid and spike) was efficacious in alleviating the disease burden as well as reducing the challenging SARS-CoV-2 virus load in the nasal passages [62]. We demonstrated here that the co-administration of S-NPs and cGAMP via the intranasal route improved not only the magnitude, but also the quality of the nasal antibody responses over immunization with S-NPs alone. This is supported by a higher level of antibodies recognizing the native structure epitopes detected in the BALs and lung homogenates of the mice that received S-NPs + cGAMP, than that of the mice receiving S-NPs alone. Despite the fact that S-NPs + cGAMP strongly induced spike- and SARS-CoV-2-specific antibody responses in the respiratory tract, we could not observe an increase in the surrogate neutralizing activity, which is detected by sVNT. This assay is designed to determine the neutralization mediated via blocking the interaction between SARS-CoV-2 RBD and the human ACE2 receptor. This inhibition, therefore, relies on the amount of anti-RBD antibodies present in each sample. We, then, concluded that intranasal immunization with S-NPs + cGAMP activated a high level of anti-spike antibody production with a low level of anti-RBD antibodies in the respiratory tract. How well this anti-spike antibody blocks the SARS-CoV-2 infection and transmission requires further study.

Besides the IgA response, a high concentration of IgG can be detected in mucosal secretions. This antibody isotype reveals a primary function similar to IgA to eliminate mucosal-invading pathogens [63]. Typically, IgG may reach the local airway space via passive diffusion through endothelial tight junctions of blood capillaries in the lower, but not the upper, respiratory tract [64,65]. The alternative is that IgG can be transcytosed across the epithelial barrier by a neonatal Fc receptor (Fcγn), which is expressed on bronchial epithelial cells [66,67]. According to our results, the predominant levels of IgG, IgG1, and IgG2a were detected in the BALs and lung tissues of S-NP-immunized mice. We also found that the presence of cGAMP enhanced IgG2a production, but not IgG1. The main function carried out by murine IgG2a over IgG1, in addition to direct agglutination, is a high affinity with the host immune components, such as complement and Fcγ-receptor-containing cells. This means that IgG2a-immune complexes potentially enhance viral clearance through antibody-mediated complement fixation and antibody-dependent cell cytotoxicity [67]. Additionally, S-NPs and cGAMP cooperatively induced mucosal IgA and IgG antibodies that bound more strongly to a WT and Delta isolate than the antibodies elicited by immunization with S-NPs. Our results suggest that intranasal immunization with a combination of S-NPs and cGAMP may confer broad immune protection. Whether the antibodies shown in our study potently block virus transmission or infection of SARS-CoV-2 WT or its variants at the nasal cavity requires further study. Unfortunately, we were unable to monitor the T cell response in NALTs. Therefore, this issue requires further investigation.

In addition to the stimulation of mucosal responses, our vaccine model also strongly activated the systemic immunities. These included the circulating IgG and IgA that exerted anti-SARS-CoV-2-neutralizing activity. Moreover, functional cytotoxic T cells and IgA^+^ B cells were markedly activated in the spleen of the mice immunized with S-NPs + cGAMP, but not the mice administered with S-NPs without cGAMP.

In conclusion, we revealed here that using TMC nanodelivery accompanied by the cGAMP adjuvant strengthened and improved the quality of immune responses against SARS-CoV-2 at the systemic and the virus-targeted organs. As most countries have been deploying booster vaccinations, our vaccine platform in the form of a nasal spray may encourage the exploitation of mucosal booster immunization. This approach is not only an atraumatic non-invasive method, but it also maximizes the induction of mucosal immunity apart from systemic responses. Indeed, the application of this platform may broaden the development of intranasal vaccines against ongoing SARS-CoV-2 variants and/or the emerging variants to come. Nevertheless, there is some concern regarding the repetitive exposure or over dosage of the intranasal vaccination because this potentially provokes immunological tolerance, resulting in unresponsiveness [68].

## Data Availability

The data presented in this study are contained within the article.

## Supplementary Materials

The following supporting information can be downloaded at: https://www.mdpi.com/article/10.3390/biomedicines10051142/s1, Figure S1: splenic cytokine profiles.
