# Supplementary material for: The STING Ligand and Delivery System Synergistically Enhance the Immunogenicity of an Intranasal Spike SARS-CoV-2 Vaccine Candidate
Source: Biomedicines. 2022 May 16;10(5):1142. doi: 10.3390/biomedicines10051142 (PMC9138454; doi:10.3390/biomedicines10051142)
Supplement: Supplementary file 1 [file biomedicines-10-01142-s001.zip › Supplementary Figure S1.pdf]

**Supplementary Figure S1**

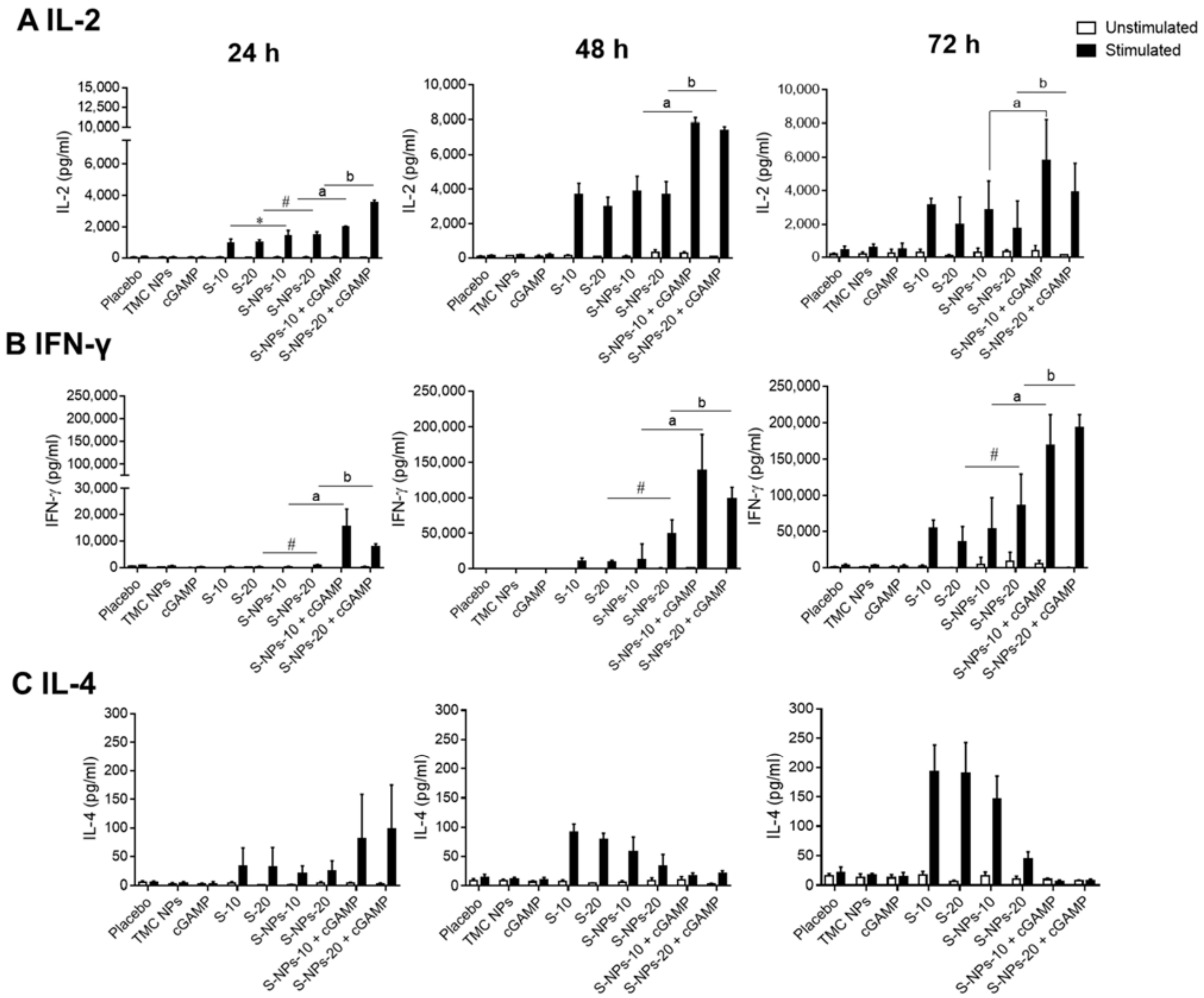


**Figure S1 Splenic cytokine profiles.** The splenocytes of immunized mice on day 45 were isolated and stimulated with S-protein (10 μg /mL). Culture supernatants were harvested for three consecutive days (24, 48, and 72 h of treatment). The levels of splenic cytokines including IL-2, IFN-γ, and IL-4 were monitored by ELISAs. Data are shown as mean ± SD (n = 4–5). * and # indicate significant differences between soluble S-protein and S-NPs at 10 and 20 μg /dose, respectively. “a” and “b” indicate significant differences of S-NPs compared with S-NPs + cGAMP at 10 and 20 μg/dose, respectively ($p < 0.05$).
